# A new panel of epitope mapped monoclonal antibodies recognising the prototypical tetraspanin CD81

**DOI:** 10.12688/wellcomeopenres.12058.1

**Published:** 2017-09-07

**Authors:** Joe Grove, Ke Hu, Michelle J. Farquhar, Margaret Goodall, Lucas Walker, Mohammed Jamshad, Heidi E. Drummer, Roslyn M. Bill, Peter Balfe, Jane A. McKeating

**Affiliations:** 1 Institute of Immunity and Transplantation, Division of Infection and Immunity, , University College London, London, NW3 2PF, UK; 2 Institute of Immunology and Immunotherapy, University of Birmingham, Birmingham, B15 2TT, UK; 3 Institute for Microbiology and Infection, School of Biosciences, University of Birmingham, Birmingham, B15 2TT, UK; 4 Centre for Biomedical Resear, Burnet Institute, Melbourne, VIC, 3004, Australia; 5 School of Life and Health Sciences, Aston University, Birmingham, B4 7ET, UK; 6 Nuffield Department of Medicine, University of Oxford, Oxford, OX3 7BN, UK

**Keywords:** tetraspanin, CD81, hepatitis C virus

## Abstract

***Background**: *Tetraspanins are small transmembrane proteins, found in all higher eukaryotes, that compartmentalize cellular membranes through interactions with partner proteins. CD81 is a prototypical tetraspanin and contributes to numerous physiological and pathological processes, including acting as a critical entry receptor for hepatitis C virus (HCV). Antibody engagement of tetraspanins can induce a variety of effects, including actin cytoskeletal rearrangements, activation of MAPK-ERK signaling and cell migration. However, the epitope specificity of most anti-tetraspanin antibodies is not known, limiting mechanistic interpretation of these studies.

***Methods**: *We generated a panel of monoclonal antibodies (mAbs) specific for CD81 second extracellular domain (EC2) and performed detailed epitope mapping with a panel of CD81 mutants. All mAbs were screened for their ability to inhibit HCV infection and E2-CD81 association. Nanoscale distribution of cell surface CD81 was investigated by scanning electron microscopy.

***Results**: *The antibodies were classified in two epitope groups targeting opposing sides of EC2. We observed a wide range of anti-HCV potencies that were independent of their epitope grouping, but associated with their relative affinity for cell-surface expressed CD81. Scanning electron microscopy identified at least two populations of CD81; monodisperse and higher-order assemblies, consistent with tetraspanin-enriched microdomains.

***Conclusions**: *These novel antibodies provide well-characterised tools to investigate CD81 function, including HCV entry, and have the potential to provide insights into tetraspanin biology in general.

## Introduction

The tetraspanin superfamily of small integral membrane proteins are characterised by their four transmembrane domains linked by intracellular and extracellular loops containing highly-conserved cysteine residues. They are present in higher eukaryotes at both the cell surface and endosomal membranes, where they exert a variety of functions, including regulating signalling, facilitating protein trafficking and influencing membrane fusion. Tetraspanins are largely without cognate ligands and are thought to function through heterotypic interactions with other membrane proteins, which they organise into so-called tetraspanin enriched microdomains or tetraspanin webs. As such, tetraspanins play an essential role in the compartmentalisation of cellular membranes ^1– 3^.

CD81, like many other tetraspanins, interacts with diverse partners in a cell type dependent manner to regulate a variety of processes, for example: CD81 association with CD19 regulates B cell signalling ^4– 6^; interactions with CD3 and ICAM-1 regulate the integrity of the immune synapse during T-cell activation ^7^; and, in concert with another tetraspanin CD9, CD81 plays a role in sperm-egg fusion, making it important for mammalian fertility ^8^. Notably, CD81 is co-opted during the life cycle of diverse human pathogens: it is involved in hepatitis C virus (HCV) and *Plasmodium* sporozoite ^9– 11^ invasion of hepatocytes, and also contributes to the assembly and budding of human immunodeficiency virus and influenza A virus ^12– 14^.

As a consequence of its involvement in these physiological and pathological processes, CD81 has become one of the most intensely-studied tetraspanins. It is, therefore, unsurprising that it is the first tetraspanin for which a complete crystal structure is available ^15^. Zimmerman *et al.* reported that the four transmembrane domains of CD81 form a cone shape containing an internal cavity. The structure revealed a single cholesterol molecule sat in this cavity, stabilised by a hydrogen bond to a closely opposed transmembrane domain. The second extracellular loop (EC2) sits across the transmembrane cone in a closed conformation. However, molecular dynamic simulations suggest that if cholesterol is removed from the central cavity of CD81, the EC2 has a propensity to switch to an open conformation; this implies that cholesterol may act as an allosteric regulator of CD81 conformation and function. It is possible that the conformation revealed in this crystal structure and the apparent cholesterol binding may be an artefact of lipid cubic phase crystallization ^15^. However, there is a well-established literature on the role of cholesterol in tetraspanin biology and more specifically on CD81-dependent cell invasion by HCV and *Plasmodium* sporozoites ^16– 19^.

Our principal interest in CD81 is in the context of HCV entry. Direct interaction between the major viral glycoprotein E2 and CD81 EC2 is essential for HCV invasion of hepatocytes ^9, 20– 22^. CD81 plays a role in the assembly of higher-order entry receptor complexes that direct HCV particles for clathrin-mediated endocytosis ^23– 25^ and fusion in the early endosome ^26^.

In the present study, we generated a panel of murine monoclonal antibodies (mAbs) against full-length CD81 to further examine these processes. Although a number of CD81 mAbs are available, little or no epitope mapping data exists ^27– 31^. We used linear peptide arrays and defined EC2 mutants to epitope map the mAbs and assessed their ability to inhibit or neutralize HCV infection. We observed a significant correlation between mAb neutralizing activity and affinity for CD81 expressed in the context of mammalian cells that was independent of epitope reactivity. Finally, we selected two high-affinity mAbs to examine the nanoscale distribution of CD81 by immunogold scanning electron microscopy (SEM); these data suggest that at least two populations of cell surface CD81 exist with distinct spatial distributions. These mAbs provide a panel of well-characterised tools to investigate the basic biology and function of CD81.

## Methods

### Cell lines, antibodies, and reagents

Huh-7.5 cells (provided by Charles Rice, The Rockefeller University, New York, NY) ^32^, Huh-7 KO CD81 (provided by Yoshiharu Matsuura, Osaka University) ^33^, Parental HepG2 and those transduced to stably express human or mouse CD81 ^34^, and 293T cells (American Type Culture Collection, ATCC) were propagated in Dulbecco’s modified Eagle medium (DMEM) supplemented with 10% foetal bovine serum and 1% nonessential amino acids (Thermo Fisher, USA). All cells were grown in a humidified atmosphere at 37°C in 5% CO _2_. Anti-NS5A mAb 9E10 was provided by C. Rice, (Rockefeller University). Rat anti-E2 antibodies 6/1a, 7/59, and 7/16 have been previously described ^35^. Secondary goat anti-mouse immunoglobulin G (IgG) antibodies, labelled with Alexa Fluor 488 (A-11001) and Alexa Fluor 647 (A-21235), was obtained from Thermo Fisher, HRP-conjugated sheep anti-mouse IgG (NA931) and goat anti-rat (NA935) was obtained from GE Healthcare.

### Generation of CD81 antibodies

Balb/c mice were immunised with recombinant human CD81 (CD81 _FL_), purified by detergent extraction from a membrane fraction of *Pichia pastoris* as previously described ^36^. Hybridomas were generated by a method based on that reported by Galfre and Milstein ^37^. NS0 immortal fusion partner cells were fused with splenocytes by PEG (StemCell Technologies, Canada). Hybridoma supernatants were screened for reactivity with CD81 _FL _and a truncated form of CD81 comprising EC2 fused to maltose binding protein (MBP-CD81 _EC2_) ^38, 39^.

### Assessing antibody interaction with CD81 by ELISA

Immulon 2HB plates (Thermo Fisher, USA) were coated with PBS containing either 5µg/mL recombinant CD81 _FL_ or a panel of MBP-CD81 _EC2_ mutant constructs, as previously described ^38, 39^. Alternatively, plates were coated with *P. pastoris* protoplast membranes containing CD81 ^36^. Overlapping peptides (25 aa, overlap of 10) corresponding to the entire sequence of CD81 were bound to plates overnight (25 µg/mL in a 50mM carbonate-bicarbonate buffer, pH9.6) ^31^. After washing and blocking with 5% BSA/PBS for 1h, the anti-CD81 mAbs were added at 2 μg/mL in PBS containing 5% BSA and 0.05% Tween-20. After a 2h incubation at room temperature, the plates were washed and bound antibody was detected with 1/1000 anti-mouse Ig-HRP, 1h incubation at room temperature. After washing, HRP-conjugates were detected colorimetrically with a TMB substrate solution and absorbance read at 450nm in accordance with the manufacturer’s instructions (BioFX, USA).

### Genesis of protoplasts expressing CD81

Recombinant CD81 protein was produced in *P. pastoris* X33 cells (Thermo Fisher) under the control of the *AOX1* promoter using the pPICZB vector (Thermo Fisher), as previously described ^36, 40, 41^. To generate protoplasts, a mid-logarithmic phase aliquot of *P. pastoris* X33 cells was re-suspended in a phosphate buffer (50mM KH _2_PO _4_, 40 mM β-mercaptoethanol, pH 7.2) and incubated for 30 min at 30°C. The cell suspension was diluted 1:1 in phosphate buffer containing 2.4 M sorbitol and Zymolyase 20T at a final concentration of 1 mg/mL and incubated for 90 min at 30°C. After harvesting, the protoplasts were washed once in phosphate buffer and resuspended in storage buffer 250mM KCl, 10mM CaCl _2_, 5mM MgCl _2_, 5mM MES, pH 7.2 supplemented with 1% glucose.

### Assessing anti-CD81 mAb binding to cells by flow cytometry

Anti-CD81 were incubated at increasing concentrations with Huh-7.5, HepG2, HepG2-CD81 cells at 37°C for 1h, in the presence of 0.01% sodium azide to prevent antibody internalization. Cells were washed 3 times in PBS and bound mAbs detected with 1/1000 Alexa Fluor 488 conjugated anti-mouse antibody, 1h at 37°C. After 3 PBS washes, cells were fixed with 1% formaldehyde and the captured fluorescent antibody quantified by flow cytometry. To acquire data, voltages for the Alexa Fluor 488 channel were set using HepG2 cells, which do not express CD81, as a negative control. To analyse, intact singlet cells were gated using the forward and side scatter channels and their median fluorescence intensity in the green channel used to assess antibody binding. Analysis was performed using FlowJo v8.3 (Treestar, USA). Representative histograms of fluorescence intensities are provided in Supplementary Figure 1.

### Genesis of virus and neutralisation of infection

Cell culture proficient HCV (HCVcc) strain J6/JFH was generated as previously described ^21^. Briefly, RNA was transcribed from linearized full-length genomes and electroporated into Huh-7.5 cells. High-titre stocks were generated by passage of the virus in naïve Huh-7.5 cells ^42^. Supernatants were collected at 72 and 96h post infection, pooled and stored at -80°C. To measure the neutralizing capacity of the anti-CD81 mAbs, Huh-7.5 cells were seeded at 1.5x10 ^4^ cells/cm ^2 ^and after 24h incubated with an increasing concentration of CD81 mAbs for 1h at 37°C, prior to infecting with HCVcc for 1h. Unbound virus or antibody was removed by washing and the media replaced with DMEM/3% FBS. After 48h, infected cells were detected after methanol fixation by staining for NS5A using the 100ng/ml 9E10 antibody, for 1 hour at room temperature; bound antibody was detected with 1/1000 Alexa Fluor 488 conjugated anti-mouse IgG, for 1 hour at room temperature, and NS5A expressing cells enumerated manually by fluorescence microscopy. mAb neutralization is defined as the percentage reduction in NS5A expressing cells compared to an irrelevant IgG. The concentration of mAb able to reduce the frequency of HCV NS5A expressing cells by 50% was calculated (IC _50_).

### Immunogold SEM and analysis

1x10 ^5^ HEK 293T were seeded onto 9mm glass coverslips and fixed after 48h in 2% EM grade formaldehyde/0.1% glutaraldehyde/PBS. Samples were quenched with 2mg/ml NaBH _4_/PBS for 5 min and blocked in 1% BSA/PBS for 30 min. The fixed cells were incubated at room temperature for 1h with anti-CD81 mAbs 1s337 or 2s20 at 2µg/ml in 1% BSA/PBS plus 0.1% BSA-c (Aurion, Netherlands), followed by 3 x 5 min PBS washes. Bound antibody was detected by incubation at room temperature for 1h with protein A conjugated to 10nm gold particles (Utrecht University Medical Center, Netherlands), diluted 1/60 in PBS + 1% BSA, followed by 3 x 5 min PBS washes.

To prepare for SEM, the samples were sequentially post-fixed in 1% glutaraldehyde, and 1% osmium/1.5% potassium ferrocyanide, and then dehydrated with serial incubation in 70, 90 and 100% ethanol. The samples were critically point dried, mounted for SEM and coated with carbon for 30 min. Samples were imaged on a Jeol 7401 high resolution Field Emission Scanning Electron Microscope (Jeol, Japan) at 40,000X magnification. To acquire images, the secondary electron channel was used to identify areas that had no topological features apparent, this mitigates the possibility of artifactual gold particle clustering due to underlying 3D structure. The distribution of gold particles was then acquired using the backscatter channel; this captures the composition of the sample and, provides maximum contrast between the cell surface and the gold particles. To quantify the images, the xy co-ordinates of each gold particle were extracted in ImageJ v2.0.0-rc-43 ^43, 44^: first the spot enhancing filter was used to enhance gold particle contrast, then gold particles were identified automatically using the ParticlePicker plugin (The New Mexico SpatioTemporal Modeling Center), with each particle manually confirmed and corrected as necessary. The particle co-ordinates were then analysed using Ripley’s L function, implemented in Matlab vR2013a (MathWorks, USA). 

### Fluorescence microscopy

Huh-7 CD81 KO cells were transduced with lentiviral vectors encoding human CD81, the transfer vector plasmid used for this can be found on Addgene. After 48h the transduced cells were seeded into 96 well plate at 1.5x10 ^4^ cells/well, alongside untransduced control cells. 24h later the cells were fixed with 4% formaldehyde and blocked in 5% BSA/PBS for 1h. Anti-CD81 hybridoma 1s73, 2s66 and 1s337 supernatants were diluted ¼ in 0.5% BSA/PBS and incubated with cells for 1h at room temperature, after washing with PBS, bound mAbs were detected with Alexa Fluor 647 conjugated anti-mouse antibody diluted 1/1000 for 1h at room temperature. At this stage 2 μg/mL DAPI was included to counterstain nuclear DNA. Samples were imaged using a Nikon Ti inverted microscope fitted with a motorized encoded stage. A 2.5 mm by 2.5 mm area of each well was acquired by image stitching using an ORCA Flash 4 sCMOS camera (Hamamatsu, Japan), with 405 nm and 647 nm fluorescence illumination provided by a PE4000 LED (CoolLED, UK) unit through a multi-band excitation/emission filter cube (Semrock, USA).

### HCV E2-CD81 binding assay

GST-CD81 _EC2_^34^ was diluted in PBS at 5ug/ml and used to coat Immulon ELISA plates overnight at 4°C. Unbound protein was removed by 3x PBS washes and wells blocked with 5% BSA/PBS for 1h at room temperature. Plates were incubated with 1µg/ml anti-CD81 mAbs or irrelevant mouse Ig and a saturating amount of soluble HCV E2 diluted in binding buffer (5% BSA/ 20% sheep serum/ 0.05% Tween 20/PBS) for 4h at room temperature. After washing, bound HCV E2 was detected using rat anti-E2 antibodies 6/1a, 7/59, and 7/16, each diluted 1:10 in binding buffer for 1h at room temperature, followed by 1/1000 HRP-conjugated anti-rat IgG, 1h incubation at room temperature. After washing, HRP-conjugates were detected colorimetrically with a TMB substrate solution and absorbance read at 450nm in accordance with the manufacturer’s instructions (BioFX, USA). Inhibition of HCV E2-CD81 association was determined relative to binding in the presence of an irrelevant mouse IgG ^34^.

## Results

### Generation of anti-CD81 monoclonal antibodies

We previously reported the expression and purification of full-length human CD81 (hCD81 _FL_) in *Pichia pastoris* ^36^. Detergent-extracted CD81 was used to immunize mice and this elicited a polyclonal antibody response in all cases. Hybridomas were generated by PEG-mediated fusion of splenocytes with the NS0 cell line and the resulting antibodies screened for reactivity with hCD81 _FL_ and a truncated form of CD81, comprising the EC2 fused to maltose binding protein (MBP-CD81 _EC2_) ^38^. Thirty-two hybridomas bound hCD81 _FL_ and 14 were reactive with MBP-CD81 _EC2_ ( Table 1). All of the reactive hybridomas were single cell cloned, isotyped and their reactivity for CD81 confirmed. To evaluate whether the mAbs recognise cell-surface CD81, we screened the panel for reactivity with HepG2 hepatoma cells that lack CD81 and with cells transduced to express human CD81 ^34^. All of the EC2-specific mAbs bound to HepG2-hCD81 cells with varying intensities, whereas the remaining non-EC2 mAbs showed negligible binding ( Table 1). None of the mAbs exhibited binding to HepG2 cells expressing murine CD81 ^34^ by indirect immunofluorescence microscopy, indicating good species specificity (data not visualised). The 18 non-EC2-specific mAbs bound to HepG2-hCD81 following fixation and permeabilization, suggesting reactivity with intracellular or transmembrane domains (data not visualised). Representative flow cytometry histogram plots for mAb binding to Huh-7.5 cells are shown in Supplementary Figure 1, alongside fluorescence microscopy images of antibody reactivity for Huh-7 CRISPR Cas9 CD81 KO cells ^33^ with and without CD81 addback, providing additional data on the specificity of these mAbs for CD81. Although not conclusive, these observations suggest that we failed to isolate mAbs targeting the smaller EC1 extracellular loop; notably, this region was not resolved in the recent crystal structure, presumably because it is structurally disordered ^15^, which may explain its poor immunogenicity.

**Table 1.** Generation of anti-CD81 monoclonal antibodies. A screen of candidate hybridomas identified thirty-two CD81 reactive clones, as summarised here. The first two columns display mAb reactivity with recombinant hCD81 _FL_ and MBP-CD81 _EC2_. The latter columns display binding to parental HepG2 cells that lack CD81 and those transduced to express exogenous CD81.

	Clone ID	Reactivity with ^1^		Binding to ^2^	
		hCD81 _FL_	MBP-CD81 _EC2_	HepG2	HepG2 _CD81_
1	**1s5**	0.71	-	-	-
2	**1s42**	0.58	-	-	-
3	**1s90**	0.68	-	-	-
4	**1s94**	0.79	-	-	-
5	**1s112**	0.87	-	-	-
6	**1s116**	1.1	-	-	-
7	**1s141**	0.67	0.06	-	-
8	**1s204**	0.79	-	-	-
9	**1s346**	0.21	-	-	-
10	**2s1**	1.16	-	-	-
11	**2s4**	0.92	-	-	-
12	**2s22**	0.89	-	-	-
13	**2s25**	1.16	-	-	-
14	**2s84**	0.98	-	-	-
15	**2s107**	0.64	-	-	-
16	**2s113**	0.32	-	-	-
17	**2s116**	1.09	-	-	-
18	**2s136**	0.77	-	-	-
19	**1s73**	0.25	0.76	-	+
20	**1s135**	1.9	1.79	-	+
21	**1s201**	2.67	2.15	-	++
22	**1s262**	0.33	0.89	-	+
23	**1s337**	1.72	1.89	-	+++
24	**2s20**	1.33	1.77	-	+++
25	**2s24**	1.9	0.45	-	+
26	**2s48**	2.33	2.1	-	++
27	**2s63**	1.53	2.01	-	+
28	**2s66**	1.76	1.72	-	++
29	**2s131**	2.01	1.94	-	++
30	**2s139**	1.57	1.99	-	++
31	**2s141**	2.13	2.02	-	+
32	**2s155**	2.7	2.06	-	+

^1^ mAb (1 μg/mL) ELISA reactivity with hCD81 _FL_ and MBP-CD81 _EC2_, where the data are expressed at optical density at 450nm. ^2^ mAb (1 μg/mL) binding to HepG2 and HepG2 _CD81_ cells, where the data represent flow cytometry median fluorescence intensity (MFI) values: - no binding; + represents an MFI < 100; ++ represents an MFI 100 – 400 and +++ represents an MFI >400.

**Figure 1.**
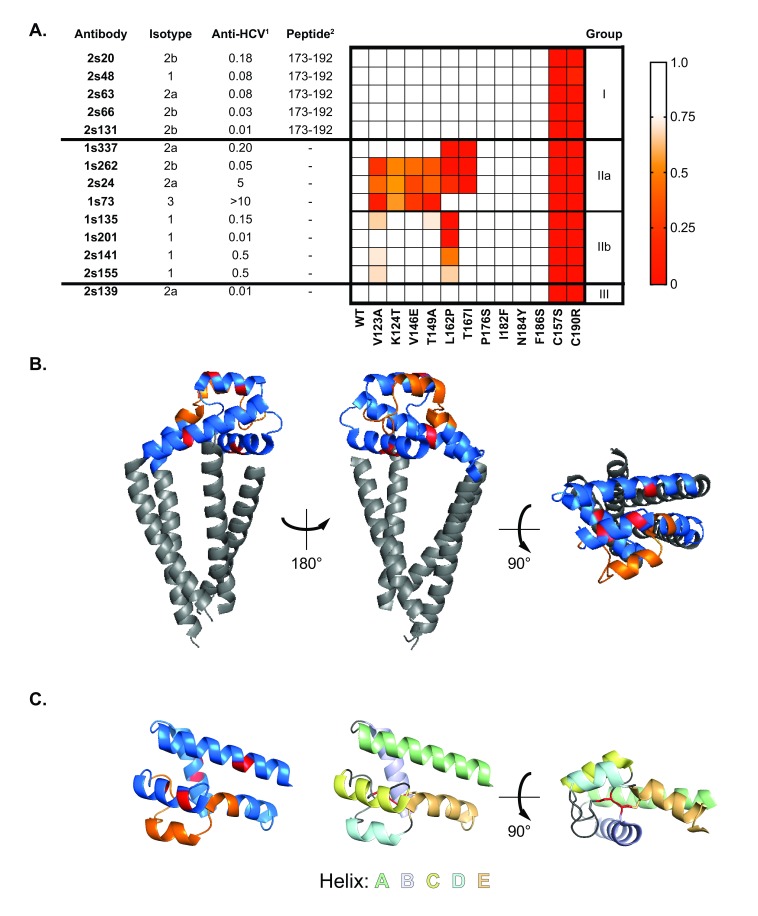
Epitope grouping of anti-CD81 EC2 mAbs. The panel of CD81 EC2-specific antibodies were epitope mapped by measuring immunoreactivity to linear overlapping peptides covering EC2 and a panel of MBP-CD81 _EC2 _constructs bearing defined point mutations. **A**. The antibody name, isotype and anti-HCV neutralizing activity are listed on the left. Reactivity against linear peptides is provided in the central column. The heat map displays reactivity against MBP-CD81 _EC2 _mutants where the data are expressed relative to wild-type MBP-CD81 _EC2_. Red colouring indicates reduced binding, as shown in the legend. The antibodies were classified into three groups according to these combined data; this is shown on the right. **B**. Relevant domains and mutations mapped onto the CD81 crystal structure (PDB: 5TCX). The EC2 is shown in blue, whilst the transmembrane domains are grey. The region binding group I mAbs is depicted in orange, whereas the mutations that block group II mAbs are shown in red. **C**. The EC2 structure is shown in isolation. The leftmost image uses the same colour coding as above, the right hand images illustrate helices A–E, as indicated in the legend. The intermolecular disulphide bonds (disrupted by the C157S and C190R mutations) are shown in red. ^1^ Anti-HCV activity is expressed as the IC _50_ (µg/ml) against HCVcc strain J6/JFH infection. ^2^ mAb (5 µg/mL) binding to overlapping EC2 peptides; reactivity was only observed for a peptide representing amino acids 173-192.

### Epitope mapping anti-CD81 EC2 mAbs

The EC2-specific mAbs were screened for their ability to bind linear peptides covering the full length of the EC2 ^31^. Five of the fourteen mAbs (2s20, 2s48, 2s63, 2s66 and 2s131) bound a peptide representing amino acids 173-192 ( Figure 1A). Attempts to localise the binding site of the mAbs to shorter peptides covering this region were unsuccessful. To further investigate epitope specificity, we exploited a panel of MBP-CD81 _EC2 _proteins bearing a variety of mutations that play a role in binding HCV E2 and that are reported to modulate CD81 conformation ^31, 38, 39^. mAb binding was assessed by ELISA and the data expressed relative to reactivity against wild type MBP-CD81 _EC2_, shown in the heat map in Figure 1A.

Mutation of cysteine residues necessary for intra-molecular di-sulphide bonds prevented the binding of all mAbs, suggesting that ‘native’ folding of EC2 is necessary for the presentation of all epitopes. A collection of mutations around helix D and E of the EC2 (residues 176, 182, 184, 186) did not affect the binding of any mAbs, despite these residues being located within the linear peptide described above. We identified a constellation of mutations, within helices A, B and C of the EC2, that abolished the reactivity of a subset of mAbs ( Figure 1A).

These data allowed us to classify the mAbs into three epitope groups: group I mAbs (2s20, 2s48, 2s63, 2s66 and 2s131) recognise a region centred around helix D of the EC2 (as identified by the peptide screen); group II mAbs (1s337, 1s262, 1s73, 1s135, 1s201, 2s24, 2s141 and 2s155) recognise similar, likely discontinuous, epitopes across helices A, B and C, and can be subdivided into groups IIa and IIb based on their response to the MBP-CD81 _EC2_ mutants; the specificity of 2s139 (designated a group III mAb) was not determined by either peptide screen or the panel of mutants. The residues that are important for group I and II mAb binding are annotated on full-length CD81 ( Figure 1B) and CD81 EC2 ( Figure 1C) structures, where the location of these regions in relation to helices A–E and critical disulphide bonds within EC2 are shown.

### Inhibition of HCV entry

HCV glycoprotein E2 binds directly to CD81 and, despite the absence of an E2-CD81 co-crystal structure, mutagenesis studies and negative stain electron microscopy have provided a reasonable understanding of this viral-receptor interaction. Current literature suggests that HCV E2 binding site for CD81 is discontinuous and is focused on a putative receptor binding loop that contacts helix D of CD81 EC2, although helix C may also contribute to binding ^38, 39, 45– 47^.

**Figure 2.**
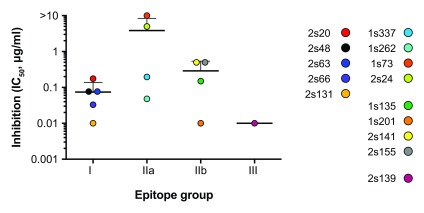
Potency of anti-HCV activity does not correlate with epitope grouping. The antibodies were assessed for their ability to inhibit HCVcc J6/JFH infection of Huh-7.5 human hepatoma cells. The data is shown as the 50% inhibitory concentration (IC _50_) for each epitope group.

Several reports suggest that anti-CD81 mAbs inhibit HCV infection by preventing this critical interaction ^27, 28^. We assessed the inhibitory activity of our anti-CD81 EC2 mAbs against HCVcc and determined the concentration of each mAb required to inhibit infection by 50% (IC _50_). Since group I mAbs target helix D, the main target for HCV E2 interactions, whereas group II antibodies appear to bind the opposing side of the EC2, we reasoned that the epitope groupings of the mAbs may define their neutralizing capacity. Figure 2 shows the IC _50_ values for each mAb grouped according to epitope specificity. Whilst the individual mAbs differ widely in their neutralizing activity (across a ~500-fold range), this did not correlate with epitope grouping. This suggests that any EC2-specific mAb has the potential to perturb CD81 interaction with HCV. To examine this further, we assessed the ability of the mAbs to inhibit binding of a truncated soluble form of HCV E2 to CD81 EC2 ( Supplementary Figure 2). All of the mAbs displayed potent blocking of this interaction, but this did not correlate with epitope grouping or neutralizing activity, suggesting that *in vitro* measurements of interactions between recombinant forms of HCV E2 and CD81 do not recapitulate the nuances of virus-receptor binding at the cell surface.

**Figure 3.**
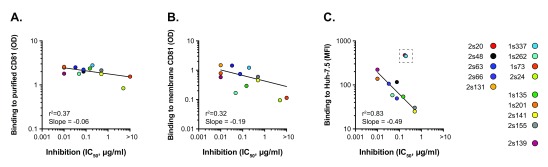
Potency of HCV entry inhibition correlates with binding to Huh-7.5 cells. Each antibody was assessed for its ability to bind to CD81 presented in different contexts **A**. Recombinant full-length CD81, expressed in *Pichia pastoris* and detergent extracted. **B**. Recombinant full-length CD81 presented on the surface membrane of *P. pastoris* protoplasts **C**. Endogenous CD81 on the plasma membrane of Huh-7.5 cells. mAb binding to purified CD81 ( **A**.) and *P. pastoris* protoplasts ( **B**.) was assessed by ELISA (optical density at 450nm), mAb binding Huh-7.5 cells ( **C**.) was assessed by flow cytometry (median fluorescence intensity). In each case half-maximal binding values are displayed. Curve fitting was performed using the log-log line equation in GraphPad Prism 6.0, the goodness of fit (r ^2^) and slope are provided for each fit. The outlying values boxed in C. were excluded from the analysis.

To further examine the correlates of mAb anti-viral activity, we assessed mAb binding to CD81 presented in three different contexts: i) recombinant full-length CD81 expressed and detergent purified from *P. pastoris* yeast, ii) membrane resident CD81 on the surface of *P. pastoris* protoplasts and iii) endogenous CD81 present in the plasma membrane of human hepatoma Huh-7.5 cells; this latter context can be considered the most physiologically-relevant setting. Figure 3 displays the relative binding affinities of the mAbs, in each context, plotted against anti-HCV neutralizing activity. Whilst all mAbs bind to detergent-purified CD81, there was little variation in signal between them and consequently this metric does not correlate with anti-HCV activity. A similar pattern was observed for mAb binding to membrane-resident CD81 on *P. pastoris* protoplasts, albeit with a modest trend towards correlation. In contrast, the intensity of mAb binding to CD81 present on the surface of Huh-7.5 cells was predictive of anti-HCV activity ( Figure 3C). Taken together, these data suggest that the strength of the intrinsic antibody-CD81 interaction is roughly equivalent for each of the mAbs, as evidenced by the comparatively similar binding profiles for recombinant CD81 ( Figure 3A & B). However, when in a physiologically-relevant membrane (i.e. Huh-7.5 hepatoma cells), CD81 is displayed in a manner that alters epitope presentation, revealing nuances in mAb binding, which, in turn, determines the potency of anti-HCV activity.

### Investigating the nanoscale distribution of CD81

Tetraspanins act as scaffolds to organise molecular events occurring at cellular membranes. They exert this function by regulating the distribution of molecular partners across the membrane. However, this characteristic can be difficult to study: it involves relatively subtle changes to the architecture of nanoscale clusters of membrane proteins, is dynamic and is likely to be dependent on the local membrane context. As such, ultra high-resolution imaging techniques, such as electron microscopy (EM) or super-resolution fluorescence microscopy, offer some of the best tools to study tetraspanin biology, as they can reveal the molecular distribution of tetraspanins and their partners *in situ* ^6, 48, 49^. We were therefore interested to ascertain whether our anti-CD81 mAbs could reveal details of the nanoscale distribution of CD81.

**Figure 4.**
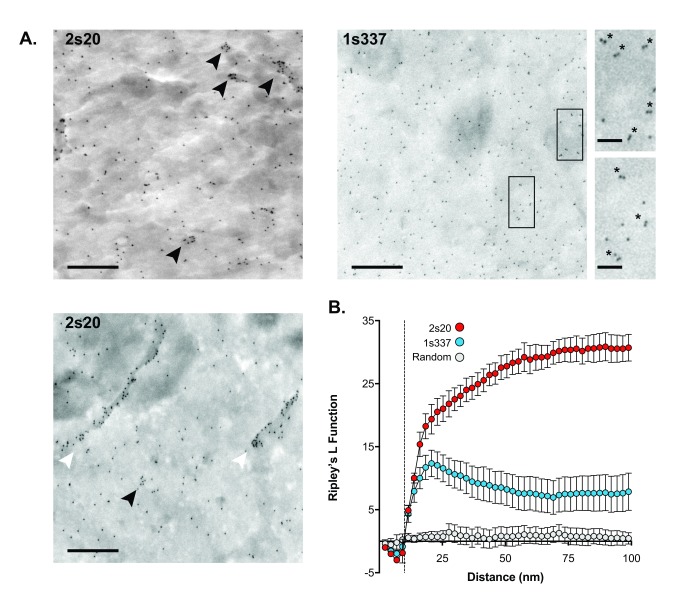
Investigation of CD81 nanoscale organisation by immunogold scanning electron microscopy. Antibodies 2s20 and 1s337 were used to assess the cell-surface distribution of CD81 on HEK 293T cells by immunogold SEM. **A**. Images display representative fields of antibody-stained cells. 2s20 staining revealed both monodisperse CD81 and CD81 organised into higher order assemblies; these included tightly packed clusters (black arrowheads) and linear arrangements (white arrowheads). 1s337 largely revealed monodispersed CD81, although there were also gold particle doublets (asterisks in inset images). Scale bars: 500nm (main images) and 100 nm (inset images). **B**. Spatial statistics analysis by Ripley’s L Function. The plot displays the calculated Ripley’s L Function values for 2s20 (n= 6 images) and 1s337 (n=14) over a 100 nm radius. A simulated random point distribution gives a straight line, as expected. Both antibodies deviate from spatial randomness, indicating clustering. For 1s337 there is a distinct peak at short distances, consistent with the gold particle doublets. 2s20 displays increasing L function values up to and beyond 100 nm, which is consistent with the higher order structures apparent in panel **A**. The dashed line indicates the smallest scale at which clustering can be assessed; this is limited by the size of the gold particles (10 nm).

Two mAbs, 2s20 (epitope group I) and 1s337 (group II), displayed high affinity for CD81 on the surface of mammalian cells ( Figure 3C). We reasoned that these would perform well in microscopy studies and used them to stain fixed cells for immunogold scanning EM. HEK 293T cells were chosen for these experiments as they express high levels of CD81 and have relatively flat plasma membranes, free of complex membrane folds that can complicate the analysis of protein distribution. Notably, HEK 293T cells can be engineered to support HCV entry, via the introduction of claudin-1 ^42^, demonstrating that CD81 is receptor active in this cellular context. Figure 4A displays representative electron micrographs of the cell surface of fixed HEK 293T cells; antibody-labelled CD81 was detected with protein A conjugated to 10nm gold particles, such that the distribution of gold particles mirrors that of CD81. Fields containing flat membrane without obvious three-dimensional membrane structures (e.g. ruffles or microvilli) were chosen to minimise artifactual clustering of gold particles.

mAb 2s20 appears to recognise two distinct populations of CD81: monodispersed CD81, represented by spatially-separated gold particles, and clustered CD81 that is arranged into higher-order assemblies. Clustered CD81 appeared in both discrete tightly-packed assemblies and in linear arrangements, the latter of which may represent accumulation of CD81 on subtle topological features ( Figure 4A). In contrast, only monodispersed CD81 was apparent in the 1s337 labelled cells, suggesting that this mAb does not bind the clustered population of CD81. However, there was a relatively high frequency of gold particle pairs, suggesting that 1s337 may detect lower order oligomers of CD81, such as dimers.

To quantify the apparent differences in mAb-labelled CD81, we used validated spatial statistics analysis tools. Ripley’s L function assesses whether the spatial organisation of a set of points, in this case gold particles, deviates from a random distribution over a range of length scales ^50– 52^. Analysis of a completely random data set generates a horizontal line, where the L function value is ~0 irrespective of the length scale ( Figure 4B). Deviation above or below this line indicates clustering or dispersal of points, respectively. The Ripley’s L function for both mAbs indicates a non-random, clustered distribution of CD81 ( Figure 4B). For mAb 2s20, the L-function increases with a gentle slope to reach a plateau at a relatively high value that extends beyond the X-axis; this indicates that 2s20-labelled CD81 exhibits clustering at length scales up to and beyond 100nm, consistent with the higher-order assemblies visible in Figure 4A. CD81 labelled with 1s337 generates a subtle curve away from randomness, with a modest peak at short length scales (~20nm), likely representing gold particle pairs, before dropping away at longer length scales, reflecting the largely monodispersed distribution of CD81. This statistical analysis is consistent with a model where at least two populations of CD81 co-exist at the plasma membrane, that are differentially recognised by the mAbs.

## Discussion

There are a variety of pre-existing mAbs targeting CD81 ^27– 30^; however, their binding specificities are unknown. Here we describe the generation of a panel of epitope-mapped anti-CD81 EC2 mAbs. The mAbs can be broadly divided in to two epitope groups: group I, which exhibit reactivity to linear EC2 peptides and likely recognise continuous epitope(s) focussed around helix D; and group II, which likely bind discontinuous epitope(s) containing elements of helixes A–C ( Figure 1C). This classification may lead us to predict that group I antibodies are relatively insensitive to protein conformation, whereas group II antibodies only recognise natively-folded EC2. However, mutational disruption of intramolecular disulphide bonds blocked the binding of all mAbs, irrespective of epitope grouping ( Figure 1A). Consistent with this, western blot analysis under reducing conditions (where disulphide bonds are broken) prevents the binding of all mAbs. Conversely, under non-reducing conditions (where disulphide bonds are intact but the protein is denatured) all group I and many group II mAbs retain reactivity. As such we have been unable to classify the mAbs as having either linear or conformational epitopes; it is likely that all mAbs require presentation of their epitope in the correct context but many tolerate some degree of protein denaturation. Representative western blots of group I and II mAbs under reducing and non-reducing conditions are provided in the OSF repository associated with this paper (see data hosting section).

It is important to note that whilst we classified the mAbs based on their apparent epitope specificity, we have not mapped the critical binding residues for any of the mAbs. For example, group I mAbs target epitope(s) contained within amino acids 173–192; nonetheless, a number of mutations within this region of EC2 (P176S, I182F, N184Y and F186S) had no effect on mAb binding ( Figure 1A), suggesting that these are not contact residues for these mAbs.

Our principal motivation for generating anti-CD81 mAbs is to study HCV entry; we tested all of the EC2 mAbs for anti-HCV activity and observed a ~500-fold range in potency ( Figure 2). Our current understanding of HCV-CD81 interactions (based on mutagenesis and structural data), suggests that E2 glycoprotein binds helix D of the EC2. This directly overlaps with the apparent target of group I mAbs and we reasoned that these mAbs may exhibit greater anti-HCV activity. However, we failed to observe any correlation between epitope specificity and anti-HCV potency ( Figure 2). This may indicate that the binding of any antibody to the EC2 is likely to prevent E2 binding; this is not unreasonable given the EC2 has a compact structure, measuring 3nm at its widest point, whereas an antibody is a bulky molecule, measuring ~15nm by 10nm. However, one of the group II mAbs (1s73) displayed negligible anti-HCV activity ( Figure 1), suggesting that antibody binding *per se* is not sufficient to prevent infection.

To further investigate the determinants of anti-HCV potency, we evaluated the binding of each mAb to CD81 expressed in a variety of contexts. We found little or no correlation between neutralizing activity and antibody binding to yeast-derived recombinant CD81 (either detergent-purified or presented on the membrane surface of *P. pastoris* protoplasts); in contrast, antibody binding to CD81 on the surface of human hepatoma cells was an excellent predictor of anti-viral potency ( Figure 3). These data suggest that expression of CD81 in more physiologically relevant membrane environments may alter EC2 presentation and/or conformation and reveal differences in mAb binding that are not apparent in *in vitro* plate based assays. Moreover, the degree of antibody binding to CD81 presented in physiological settings determines anti-HCV potency.

We have not investigated the potential compositional or biochemical differences between yeast and mammalian membranes that may influence CD81 presentation; however, there are at least two important features that should be considered. The interactions between CD81 and its various molecular partners (e.g. CD9, CD19, CD3) are poorly understood; nonetheless, it is likely that heterotypic interactions occurring in the plasma membrane of mammalian cells alters CD81 epitope availability and modulate mAb binding. Although tetraspanins are found in all higher eukaryotes they are completely absent from yeast ^53^, consequently, CD81 will not have any natural binding partners in *P. pastoris* and this may impact epitope presentation. Furthermore, one interpretation of the recent crystal structure of CD81 might be that cholesterol, resident in the plasma membrane of mammalian cells, may regulate CD81 EC2 conformation ^15^. Unlike mammalian cells, the principal sterol in yeast membranes is ergosterol ^54^, and conformational switching may not occur in this setting. Whether these mAbs can differentiate between ‘open’ and ‘closed’ conformers of CD81 is a focus of on-going investigations.

Finally, we used two mAbs as tools to study the molecular distribution of CD81. Immunogold labelling of cell-surface endogenous CD81, combined with spatial statistics analysis, identified at least two populations of CD81 with distinct distributions: monodispersed CD81 with little spatial organisation and clustered CD81 found in higher-order assemblies, possibly representing tetraspanin-enriched microdomains ^1– 3^. This data may be consistent with the notion of a dynamic equilibrium of CD81 transitioning from monomeric to oligomeric forms. Notably, the two mAbs appeared to differ in their ability to recognise these distinct states of CD81: 2s20 labelling revealed both clustered and monodispersed CD81, whereas large clusters of CD81 were not apparent on 1s337-labelled cells. Further studies will be necessary to understand the relevance of these different distributions of CD81.

Here, we present a panel of epitope-mapped and validated anti-CD81 mAbs that have immediate relevance to the study of HCV entry. Moreover, we believe they may provide useful tools for investigating the basic biology of CD81 and tetraspanins in general. The ability to study CD81 distribution *in situ*, and to discriminate different membrane-resident populations, is particularly valuable given that tetraspanin function is defined by an ability to spatially organise their molecular partners and compartmentalise cellular membranes.

## Data availability

The data referenced by this article are under copyright with the following copyright statement: Copyright: © 2017 Grove J et al.

The data underlying Figure 1– Figure 4 and S1, S2 and further supporting data are provided at the Open Science Framework http://doi.org/10.17605/OSF.IO/HKN3X ^55^

## Animal statement

The immunisation protocol and number of animals required were submitted for ethical review by the University of Birmingham Ethical Review SubCommittee (BERSC) and conducted under Home Office licence PPL 40/2220 and PIL 40/2284.

The study design required two female young adult Balb/c mice obtained from the Specific Pathogen Free (SPF) breeding unit in the University of Birmingham BioMedical Services Unit. The initial injection, no more than 100μl sub-cutaneously, as per home office guidelines, was combined with Complete Freund’s Adjuvant, which had been previously shown not to raise inflammatory reactions. Three booster immunisations of 100μl in Phosphate Buffered Saline (PBS) were administered intraperitoneally at 2 week intervals. The animals were monitored within 4h, at the end of the working day and twice daily thereafter for any adverse reactions. None were apparent. A tail bleed of not more than 50μl was taken 6 weeks after the primary immunisation and assessed for a specific polyclonal response. A chosen mouse was culled, in accordance with home office guidelines, and the spleen sterilely excised. The second mouse was culled for a fusion 2 weeks later, having received 1 more booster injection.

## Supplementary material

**Supplementary Figure 1: Representative immunostaining with anti-CD81 mAbs.** **A.** Fluorescence intensity histograms for CD81 labeled Huh-7.5 cells acquired by flow cytometry. Examples of low (1s73), intermediate (2s66) and high affinity mAbs (1s337) are provided. The grey histogram represents binding of an irrelevant mouse IgG. All mAbs used at 1µg/ml **B.** Matched microscopy images of immunostained Huh-7 CD81 KO cells with and without lentiviral transduction to add back CD81. Scale bar 200µm.

**Supplementary Figure 2: Inhibition of E2-CD81 interactions.** The binding of soluble HCV E2 to GST-CD81 _EC2_ in the presence of 1µg/ml of anti-CD81 mAb was monitored by ELISA. The percent inhibition was calculated by comparison to E2-CD81 interactions occurring in the presence of an irrelevant mouse IgG.
