# Supplementary figures and images for: A new panel of epitope mapped monoclonal antibodies recognising the prototypical tetraspanin CD81
Source: Wellcome Open Res. 2017 Sep 7;2:82. [Version 1] doi: 10.12688/wellcomeopenres.12058.1 (PMC5657224; doi:10.12688/wellcomeopenres.12058.1)

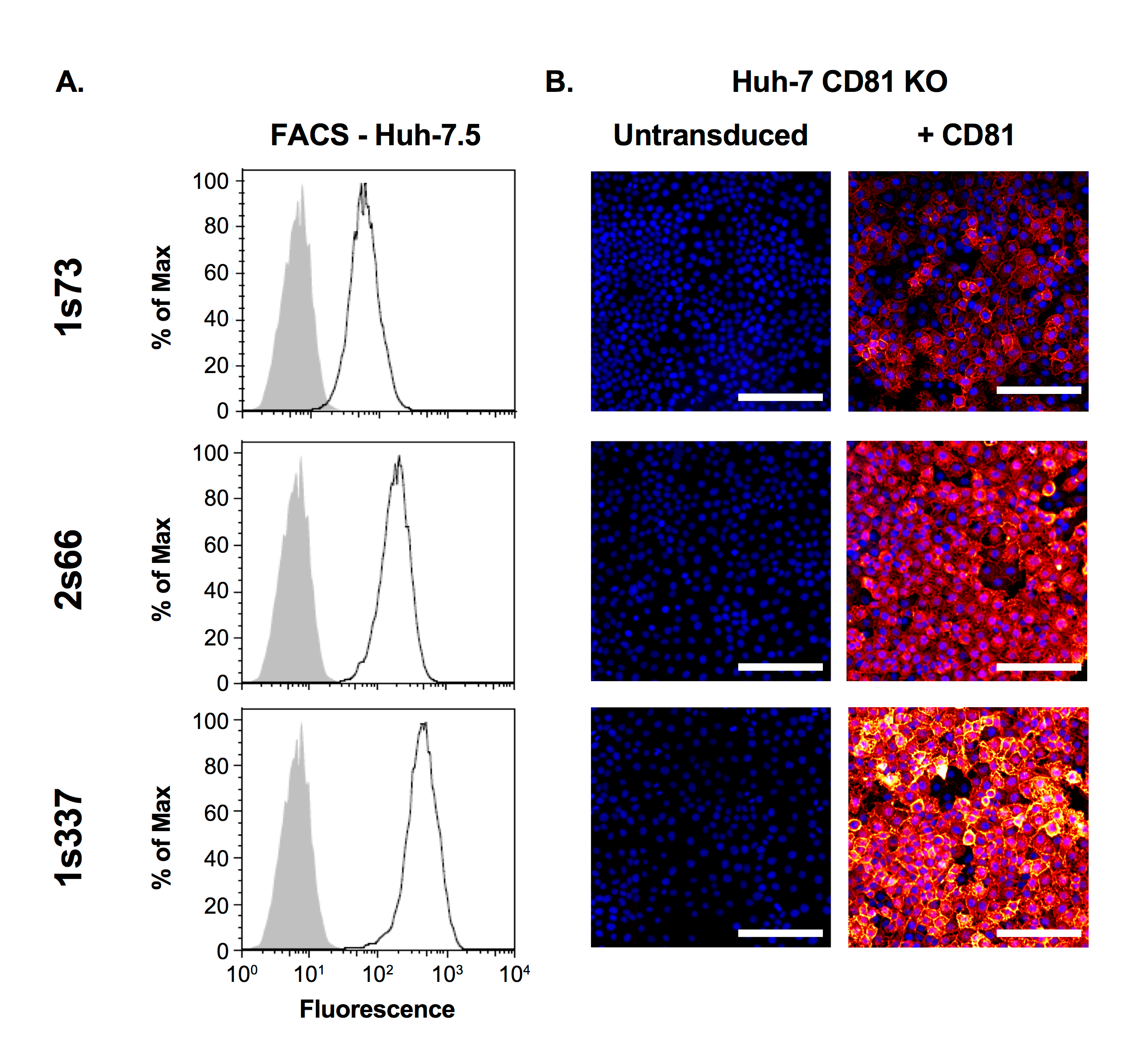

Supplement: Supplementary file 1 [file wellcomeopenres-2-13046-s0000.tgz › e37c68c4-e60d-41b3-80c1-b97a36a7e867.png]

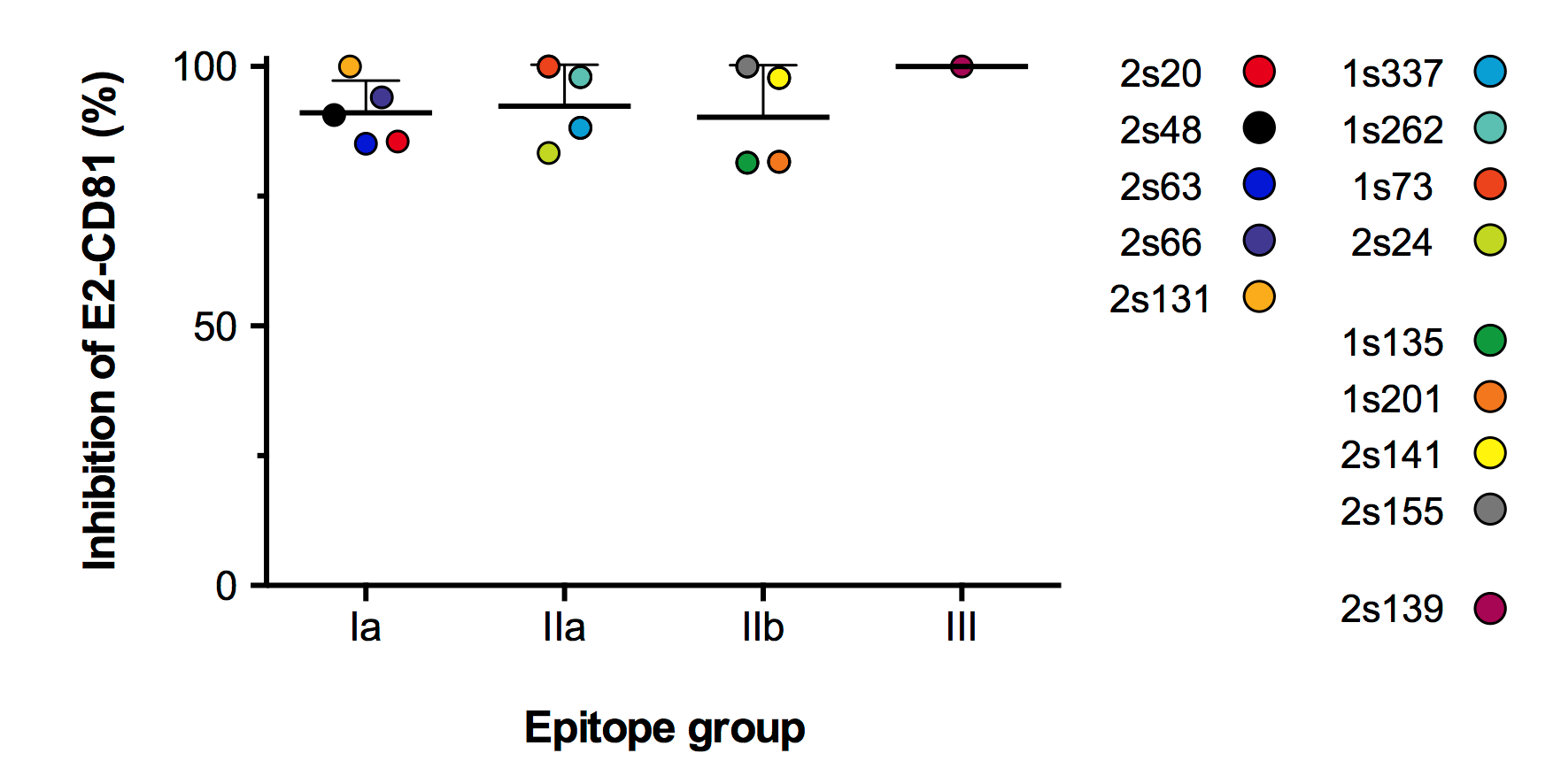

Supplement: Supplementary file 2 [file wellcomeopenres-2-13046-s0001.tgz › 69cad4d1-8620-48ef-a30c-84b3eb10dc88.png]
