# Supplementary material for: A new panel of epitope mapped monoclonal antibodies recognising the prototypical tetraspanin CD81
Source: Wellcome Open Res. 2017 Sep 7;2:82. [Version 1] doi: 10.12688/wellcomeopenres.12058.1 (PMC5657224; doi:10.12688/wellcomeopenres.12058.1)
Supplement: Peer review history

# Peer review: Referee response for version 1

**DOI:** 10.21956/wellcomeopenres.13046.r25932

**Published:** 2017-10-20

**Authors:** Stephen Griffin

**Affiliations:** 1 Faculty of Medicine and Health, St James's University Hospital, University of Leeds, Leeds, UK

Grove and colleagues detail the creation of a panel of anti human CD81 antibodies, which they intended for use characterising interactions with hepatitis C virus (HCV) during the entry process. The monoclonals have been well characterised, especially compared with other reagents available commercially, and encompass reagents specific to both the large extracellular loop and other regions of the protein.

 Select reagents were then used as competitors for HCV entry, and to stain CD81 complexes on the surface of HEK cells. The authors found no correlation between m'ab binding sites and ability to block HCV entry, but did correlate this with the overall avidity of m'abs for cell expressed CD81. Scanning immunogold EM conducted upon plasma membranes revealed apparently distinct complexes of CD81, consistent with its organisation within the tetraspannin web.

 Some comments for consideration:

1. For the HCV entry inhibition experiments, have the authors considered that entry may be blocked by the m'abs promoting internalisation of the CD81? This may explain the apparent independence with respect to binding sites.
2. I do not understand the statistical basis for ignoring the outliers in figure 3c. Plus experimental repeats and tests should be clarified in general.
3. Controls are missing for the SEM studies, such as primary/secondary antibody controls and cells lacking CD81, such as HepG2.

I have read this submission. I believe that I have an appropriate level of expertise to confirm that it is of an acceptable scientific standard, however I have significant reservations, as outlined above.

# Peer review: Referee response for version 1

**DOI:** 10.21956/wellcomeopenres.13046.r25809

**Published:** 2017-09-26

**Authors:** Peter N. Monk

**Affiliations:** 1 Department of Infection, Immunity & Cardiovascular Disease, University of Sheffield, Sheffield, UK

The article discusses the ability of a set of antibodies raised against CD81 expressed in Pichia pastoris to inhibit hepatitis C virus entry. The antibodies may be useful research tools in further defining the role of CD81 in viral entry, and the functions of tetraspanins generally. Generally, the experiments have been performed well, and the article is clearly written. However, there are some points that should be addressed by the authors:

1. Antibody inhibition of HCV binding is presumed to be by steric hindrance rather than blockade of specific binding sites. This could be explored more clearly using fab fragments of the antibodies that are a fraction of the size of the intact antibodies. Why was this simple and obvious procedure not performed?
2. The hybridoma supernatants were screened against a recombinant form of the CD81 EC2 fused to MBP. It is well know that recombinant tetraspanin EC2 proteins contain a proportion of misfolded protein that could bias the selection of hybridomas. Why was screening not primarily performed against a cell line expressing native CD81?
3. GST-CD81 EC2 was used for evaluation of HCV E2 binding. Why was this used rather than MBP-EC2?
4. No competitive binding study was performed. This could have helped define EC2 domains recognised by the antibodies, particularly if fab fragments were used.
5. In Fig 3c, the authors decide to ignore 2 outliers to perform the linear regression analysis. On what statistical basis were these 'outliers' defined? What are the biological properties associated with these antibodies that might make them behve in this way?
6. The selection of antibodies for nanoscale distribution experiments was performed on the basis of high affinity. A more rational choice for this might have been one high and one low affinity antibody. Low affinity antibodies have been used by several research groups to discriminate clustered v unclustered tetraspanins at the cell surface.
7. Although the authors detect apparently different populations of CD81 on HEK cells, it is very difficult to draw any conclusions from a single type of experiment. Does modulation of cholesterol or the actin cytoskeleton affect the populations? Does clustering of tetraspanin-associated proteisn affect distribution? Do the different populations bind EC2 differently?

I have read this submission. I believe that I have an appropriate level of expertise to confirm that it is of an acceptable scientific standard, however I have significant reservations, as outlined above.

# Peer review: Referee response for version 1

**DOI:** 10.21956/wellcomeopenres.13046.r25963

**Published:** 2017-09-21

**Authors:** Annemiek B. van Spriel

**Affiliations:** 1 Radboud Institute for Molecular Life Sciences, Radboud University Medical Centre, Nijmegen, Netherlands

This manuscript documents the generation and characterization of novel monoclonal antibodies (mAbs) specific for tetraspanin CD81. The antibodies were classified in two epitope groups targeting opposing sides. A wide range of anti-HCV potencies was observed independent of their epitope grouping, but associated with their relative affinity for cell-surface expressed CD81. In addition, testing the CD81-antibodies in SEM revealed both monomeric and clustered CD81 molecules.

 The manuscript is well-written and the novel CD81-antibodies provide new tools to study CD81 and HCV binding.

 Suggestions for improvement:

- It is observed that the group I mAbs (2s20, 2s48, 2s63, 2s66 and 2s131) recognise a region centered around helix D of the EC2. In this context and in relation to their SEM data, it is important to discuss the role of the delta-helix in CD81 dimerisation ^1^.
- The current SEM analyse does not include antibody isotype controls. These should be included to exclude aspecific signals in SEM analysis.
- Super-resolution of the tetraspanin web has recently revealed that tetraspanins (including CD81) preferentially homodimerize ^2^. The discussion may benefit from reflecting the current SEM data with this study.
- CD81 also interferes with HIV replication. Did the authors also analyse the novel CD81-antibodies in respect to blocking HIV antigen binding or replication? This should at least be discussed.
- It is unclear why the 1s337 antibody only recognizes mononeric CD81. Can the authors speculate what the underlying mechanism is? Is it interference with a CD81-partner binding?
- Include statistics, in particular how many times experiments have been performed.

I have read this submission. I believe that I have an appropriate level of expertise to confirm that it is of an acceptable scientific standard.

## References

[1] SchmidtTHHomsiYLangT: Oligomerization of the Tetraspanin CD81 via the Flexibility of Its δ-Loop. *Biophys J*.2016;110(11) : 10.1016/j.bpj.2016.05.003 2463-2474 10.1016/j.bpj.2016.05.003 27276264PMC4906359

[2] ZuidscherwoudeMGöttfertFDunlockVMFigdorCGvan den BogaartGvan SprielAB: The tetraspanin web revisited by super-resolution microscopy. *Sci Rep*.2015;5: 10.1038/srep12201 12201 10.1038/srep12201 26183063PMC4505338
